# Supplementary material for: Multi‐dataset identification of innovative feature genes and molecular mechanisms in keratoconus
Source: J Cell Mol Med. 2024 Sep 19;28(18):e70079. doi: 10.1111/jcmm.70079 (PMC11412914; doi:10.1111/jcmm.70079)
Supplement: Supplementary file 2 — Material S2. [file JCMM-28-e70079-s002.zip › 7.GSEA analysis/2.ATOH7/KEGG_FC_GAMMA_R_MEDIATED_PHAGOCYTOSIS.html]

Details for gene set KEGG\_FC\_GAMMA\_R\_MEDIATED\_PHAGOCYTOSIS[GSEA]

|  || Dataset | ATOH7.group.cls#h\_versus\_l |
| Phenotype | group.cls#h\_versus\_l |
| Upregulated in class | h |
| GeneSet | KEGG\_FC\_GAMMA\_R\_MEDIATED\_PHAGOCYTOSIS |
| Enrichment Score (ES) | 0.3649972 |
| Normalized Enrichment Score (NES) | 1.4765245 |
| Nominal p-value | 0.06439394 |
| FDR q-value | 0.2654659 |
| FWER p-Value | 0.816 |
Table: GSEA Results Summary

  

Fig 1: Enrichment plot: KEGG\_FC\_GAMMA\_R\_MEDIATED\_PHAGOCYTOSIS      
 Profile of the Running ES Score & Positions of GeneSet Members on the Rank Ordered List

  

| SYMBOL | TITLE | RANK IN GENE LIST | RANK METRIC SCORE | RUNNING ES | CORE ENRICHMENT || 1 | PIP5K1B | na | 2 | 1.293 | 0.1079 | Yes |
| 2 | AKT3 | na | 9 | 1.043 | 0.1948 | Yes |
| 3 | PRKCE | na | 1002 | 0.447 | 0.2035 | Yes |
| 4 | ASAP1 | na | 1030 | 0.443 | 0.2397 | Yes |
| 5 | AMPH | na | 1471 | 0.389 | 0.2595 | Yes |
| 6 | VAV1 | na | 1743 | 0.365 | 0.2822 | Yes |
| 7 | GAB2 | na | 2148 | 0.333 | 0.2983 | Yes |
| 8 | PRKCB | na | 2281 | 0.324 | 0.3216 | Yes |
| 9 | PIP5K1A | na | 3253 | 0.267 | 0.3158 | Yes |
| 10 | DOCK2 | na | 3349 | 0.262 | 0.3350 | Yes |
| 11 | MARCKSL1 | na | 3549 | 0.254 | 0.3504 | Yes |
| 12 | MAP2K1 | na | 4240 | 0.228 | 0.3495 | Yes |
| 13 | CFL2 | na | 4649 | 0.213 | 0.3555 | Yes |
| 14 | PAK1 | na | 4910 | 0.204 | 0.3650 | Yes |
| 15 | CRKL | na | 5867 | 0.175 | 0.3520 | No |
| 16 | PIK3R3 | na | 6166 | 0.166 | 0.3572 | No |
| 17 | LYN | na | 6447 | 0.158 | 0.3624 | No |
| 18 | NCF1 | na | 7020 | 0.143 | 0.3578 | No |
| 19 | WASF3 | na | 7566 | 0.129 | 0.3528 | No |
| 20 | PRKCA | na | 7938 | 0.120 | 0.3521 | No |
| 21 | SCIN | na | 8338 | 0.112 | 0.3499 | No |
| 22 | FCGR3A | na | 8648 | 0.106 | 0.3498 | No |
| 23 | CRK | na | 8997 | 0.099 | 0.3480 | No |
| 24 | WAS | na | 9234 | 0.095 | 0.3491 | No |
| 25 | FCGR2B | na | 9507 | 0.090 | 0.3488 | No |
| 26 | ASAP2 | na | 9534 | 0.090 | 0.3555 | No |
| 27 | SPHK2 | na | 9717 | 0.086 | 0.3574 | No |
| 28 | ARPC1B | na | 9852 | 0.084 | 0.3605 | No |
| 29 | MARCKS | na | 10188 | 0.078 | 0.3574 | No |
| 30 | LIMK2 | na | 11319 | 0.061 | 0.3299 | No |
| 31 | DNM1 | na | 12339 | 0.048 | 0.3045 | No |
| 32 | LAT | na | 13347 | 0.037 | 0.2785 | No |
| 33 | RAC2 | na | 13916 | 0.031 | 0.2647 | No |
| 34 | DNM3 | na | 14259 | 0.028 | 0.2572 | No |
| 35 | PIP5K1C | na | 14534 | 0.025 | 0.2514 | No |
| 36 | MAPK1 | na | 14536 | 0.025 | 0.2535 | No |
| 37 | PLD1 | na | 14651 | 0.024 | 0.2522 | No |
| 38 | PLA2G4F | na | 14672 | 0.024 | 0.2537 | No |
| 39 | RAF1 | na | 15219 | 0.019 | 0.2395 | No |
| 40 | PLA2G4A | na | 15227 | 0.019 | 0.2409 | No |
| 41 | WASF1 | na | 15259 | 0.019 | 0.2416 | No |
| 42 | DNM2 | na | 15705 | 0.015 | 0.2300 | No |
| 43 | AKT1 | na | 15787 | 0.014 | 0.2289 | No |
| 44 | PLA2G6 | na | 15813 | 0.014 | 0.2293 | No |
| 45 | ARF6 | na | 15942 | 0.013 | 0.2267 | No |
| 46 | ARPC5L | na | 16114 | 0.012 | 0.2228 | No |
| 47 | PIK3CD | na | 16202 | 0.011 | 0.2212 | No |
| 48 | RAC1 | na | 16331 | 0.010 | 0.2184 | No |
| 49 | CFL1 | na | 16342 | 0.010 | 0.2189 | No |
| 50 | MAPK3 | na | 16821 | 0.006 | 0.2057 | No |
| 51 | ARPC5 | na | 16961 | 0.005 | 0.2020 | No |
| 52 | ARPC1A | na | 17138 | 0.003 | 0.1972 | No |
| 53 | ARPC2 | na | 17185 | 0.003 | 0.1961 | No |
| 54 | PLD2 | na | 17242 | 0.002 | 0.1947 | No |
| 55 | FCGR2C | na | 17670 | -0.001 | 0.1825 | No |
| 56 | WASF2 | na | 17673 | -0.001 | 0.1825 | No |
| 57 | CDC42 | na | 17874 | -0.003 | 0.1770 | No |
| 58 | PRKCD | na | 17924 | -0.003 | 0.1759 | No |
| 59 | SYK | na | 18025 | -0.004 | 0.1733 | No |
| 60 | WASL | na | 18186 | -0.006 | 0.1692 | No |
| 61 | ARPC3 | na | 19901 | -0.021 | 0.1214 | No |
| 62 | PIK3CB | na | 20685 | -0.028 | 0.1012 | No |
| 63 | VAV3 | na | 21180 | -0.034 | 0.0897 | No |
| 64 | PIK3CA | na | 21359 | -0.036 | 0.0876 | No |
| 65 | VASP | na | 21800 | -0.041 | 0.0783 | No |
| 66 | VAV2 | na | 22149 | -0.045 | 0.0719 | No |
| 67 | PIK3R2 | na | 22209 | -0.045 | 0.0740 | No |
| 68 | PIKFYVE | na | 22283 | -0.046 | 0.0757 | No |
| 69 | RPS6KB1 | na | 22694 | -0.051 | 0.0681 | No |
| 70 | SPHK1 | na | 23089 | -0.056 | 0.0614 | No |
| 71 | GSN | na | 23148 | -0.057 | 0.0645 | No |
| 72 | RPS6KB2 | na | 23175 | -0.057 | 0.0685 | No |
| 73 | MYO10 | na | 23267 | -0.058 | 0.0708 | No |
| 74 | PIP4K2B | na | 23746 | -0.066 | 0.0625 | No |
| 75 | DNM1L | na | 24243 | -0.073 | 0.0543 | No |
| 76 | PLCG2 | na | 24577 | -0.078 | 0.0512 | No |
| 77 | ARPC4 | na | 24640 | -0.080 | 0.0561 | No |
| 78 | PLCG1 | na | 25238 | -0.090 | 0.0463 | No |
| 79 | PIK3CG | na | 26210 | -0.109 | 0.0273 | No |
| 80 | LIMK1 | na | 26531 | -0.115 | 0.0277 | No |
| 81 | AKT2 | na | 27111 | -0.128 | 0.0217 | No |
| 82 | PLA2G4E | na | 27235 | -0.132 | 0.0292 | No |
| 83 | HCK | na | 27450 | -0.137 | 0.0344 | No |
| 84 | PIK3R1 | na | 27720 | -0.144 | 0.0386 | No |
| 85 | FCGR1A | na | 28474 | -0.164 | 0.0305 | No |
| 86 | PTPRC | na | 28489 | -0.164 | 0.0438 | No |
| 87 | INPP5D | na | 28637 | -0.168 | 0.0536 | No |
| 88 | PRKCG | na | 28978 | -0.178 | 0.0587 | No |
| 89 | ASAP3 | na | 29010 | -0.180 | 0.0728 | No |
| 90 | FCGR2A | na | 30291 | -0.221 | 0.0543 | No |
| 91 | PLA2G4D | na | 31570 | -0.272 | 0.0401 | No |
| 92 | PIK3R5 | na | 34383 | -0.611 | 0.0100 | No |
Table: GSEA details [plain text format]

  

Fig 2: KEGG\_FC\_GAMMA\_R\_MEDIATED\_PHAGOCYTOSIS      
 Blue-Pink O' Gram in the Space of the Analyzed GeneSet

  

Fig 3: KEGG\_FC\_GAMMA\_R\_MEDIATED\_PHAGOCYTOSIS: Random ES distribution      
 Gene set null distribution of ES for **KEGG\_FC\_GAMMA\_R\_MEDIATED\_PHAGOCYTOSIS**
